# A Modified Sine-Cosine Algorithm Based on Neighborhood Search and Greedy Levy Mutation

**DOI:** 10.1155/2018/4231647

**Published:** 2018-07-04

**Authors:** Chiwen Qu, Zhiliu Zeng, Jun Dai, Zhongjun Yi, Wei He

**Affiliations:** ^1^School of Information Engineering, Baise University, Baise 533000, China; ^2^School of Tourism Management, Baise University, Baise 533000, China; ^3^School of Business Administration, Baise University, Baise 533000, China; ^4^School of Politics and Public Affair Management, Baise University, Baise 533000, China

## Abstract

For the deficiency of the basic sine-cosine algorithm in dealing with global optimization problems such as the low solution precision and the slow convergence speed, a new improved sine-cosine algorithm is proposed in this paper. The improvement involves three optimization strategies. Firstly, the method of exponential decreasing conversion parameter and linear decreasing inertia weight is adopted to balance the global exploration and local development ability of the algorithm. Secondly, it uses the random individuals near the optimal individuals to replace the optimal individuals in the primary algorithm, which allows the algorithm to easily jump out of the local optimum and increases the search range effectively. Finally, the greedy Levy mutation strategy is used for the optimal individuals to enhance the local development ability of the algorithm. The experimental results show that the proposed algorithm can effectively avoid falling into the local optimum, and it has faster convergence speed and higher optimization accuracy.

## 1. Introduction

Many problems in the field of engineering practice and scientific research come down to the global optimization problems. The traditional methods which purely lie upon the exactly mathematical mode have unsatisfactory effect in solving such optimization problems. These problems need to be continuous and derivable when the traditional methods are used for solving such practical engineering optimization problems, and these methods do not have the ability of global optimization for the multimodal, strong-nonlinearity, and dynamic change problems [1]. Accordingly, many scholars begin to explore new solution methods. The swarm intelligence optimization algorithm is a kind of global optimization algorithm designed by simulating the mutual cooperation behavior mechanism of gregarious biology in nature. Compared with the traditional optimization methods, the swarm intelligence optimization algorithm is characterized by simple principle and fewer adjustment parameters, and the gradient information and strong global optimization algorithm of problems are not required. So it is widely used in the engineering field of function optimization [2–4], combinatorial optimization [5], neural network training [6, 7], and image processing. At present, many swarm intelligence optimization algorithms are proposed [2, 8–15] like particle swarm optimization (PSO) [8], differential evolution (DE) [9, 10], artificial bee colony algorithm (ABC) [2, 11], cuckoo search (CS) [12, 13], and flower pollination algorithm (FPA) [14, 15].

Sine-cosine algorithm (SCA) is a new swarm intelligence optimization algorithm proposed by Mirjalili in 2016 [16]. This algorithm has been concerned and studied by many scholars due to its simple implementation and less parameter setting, and its optimization search can be realized through simple variation of sine and cosine function values. It has been successfully applied to solving the parameter optimization of support vector regression [17], short-term hydrothermal scheduling [18], and other engineering fields at present. However, as with other swarm intelligence algorithms, this algorithm also has the disadvantage of low optimization precision and slow convergence speed. Many scholars have put forward various improved sine-cosine algorithms from different perspectives in order to overcome this disadvantage in last two years. Elaziz et al. [19] proposed a sine-cosine algorithm based on the opposition method, and the more accurate solutions is obtained. Nenavath et al. [20] adopted a hybrid algorithm by combining differential evolution with sine-cosine to solve the problem of global optimization and target tracking. This algorithm has faster convergence speed and ability of seeking the optimal solution compared with the basic sine-cosine algorithm and differential evolution algorithm. Reddy et al. [21] applied a new binary variant of sine-cosine algorithm to solve the PBUC (profit-based unit commitment) problem. Sindhu et al. [22] used the elitism strategy and new updating mechanism to improve the sine-cosine algorithm, which improved the accuracy of classification in the selection of features or attributes. Kumar et al. [23] proposed a new sine-cosine optimization algorithm with the hybrid Cauchy and Gaussian mutations in order to track MPP (maximum power point) quickly and efficiently. Mahdad et al. [24] presented a sine-cosine algorithm coordinated with the interactive process to improve the security of the power system aimed at loading margin stability and faults at specified important branches. Bureerat et al. [25] adopted an adaptive differential sine-cosine algorithm to solve the problem of structural damage detection. Turgut et al. [26] combined backtracking search algorithm (BSA) and sine-cosine algorithm (SCA) to obtain the optimal design for the shell and tube evaporator. Attia et al. [27] embed Levy's flight into the original sine-cosine algorithm to increase the local search ability of the algorithm and avoided the algorithm being trapped in a local optimal defect. Tawhid et al. [28] used elite nondominated sorting to obtain different nondominated grades and applied crowd distance method to maintain the diversity of optimal solution sets, putting forward a multiobjective SCA algorithm. Issa et al. [29] presented an enhanced version of SCA by embedding the particle swarm optimization algorithm in SCA(ASCA-PSO). The ASCA-PSO algorithm makes full use of developing ability of the particle swarm optimization algorithm in the search space, which is stronger than that of the SCA. In the tests of some functions, it is found that the search performance of ASCA-PSO is apparently superior to that of SCA and other recently proposed basic metaheuristic algorithms. Rizk-Allah et al. [30] proposed a multiorthogonal sine-cosine algorithm (MOSCA) based on a multiorthogonal search strategy (MOSS) to solve the problem of engineering designs. The MOSCA algorithm eliminated the disadvantages which are that the basic SCA lacked exploitability and it was easily trapped in local optimum.

The modified sine-cosine algorithm (MSCA) based on neighborhood search and the greedy Levy mutation has been proposed in order to better balance the global exploration ability and local exploitation ability. The improved algorithm makes improvements in the following three aspects. Firstly, both the linear decreasing inertia weight and exponential declining conversion parameters are used to balance the global exploration and local exploitation ability, which achieves the smooth transition of algorithm from global exploration to local development. Secondly, the guidance of random individuals near the optimal solution is fully used to allow the algorithm easily jump out of the local optimum, which effectively prevents the algorithm premature convergence and increases the diversity of population. Thirdly, the greedy Levy mutation strategy is used for the optimal individuals to enhance the local development ability of the algorithm. Compared with other swarm intelligence algorithms, the improved sine-cosine algorithm has better performance in terms of searching precision, convergence speed, and stability.

## 2. Basic Sine-Cosine Algorithm

In the basic sine-cosine algorithm, the simple variation of sine and cosine function values is used to achieve the optimization search. In this paper, the population size is* n*. The dimension of search space is* d*, and the* i*th individual in the population is *p*_*i*_. In each iteration, the update mode of *p*_*i*_ can be obtained by the following equation:

(1) pi,jt+1=pi,jt+r1·sin⁡r2·r3·pbest,jt−pi,jt,r4<0.5pi,jt+r1·cosr2·r3·pbest,jt−pi,jt,r4≥0.5

where* t* is the current iteration, *p*_*best*,*j*_^*t*^  is the* j*th dimension value of the optimal individual at iteration* t*, and *p*_*i*,*j*_^*t*^ is the* j*th dimension value of the individual* i* at iteration* t*. *r*_1_, *r*_2_, *r*_3_, and *r*_4_ are the random numbers. *r*_1_ and *r*_3_ obey uniform distribution between 0 and 2. *r*_2_ obey uniform distribution between 0 and 2*π*, and *r*_4_ obey uniform distribution between 0 and 1.

In (1), *r*_1_ · sin(*r*_2_) or *r*_1_ · cos(*r*_2_) jointly lead the global exploration and local development ability of the algorithm. When the value of *r*_1_ · sin(*r*_2_) or *r*_1_ · cos(*r*_2_) is greater than 1 or less than -1, the algorithm conducts a global exploration search. When the value of *r*_1_ · sin(*r*_2_) or *r*_1_ · cos(*r*_2_) is within the range of [-1  1], the algorithm conducts a local development search. The value of sin(*r*_2_)or cos(*r*_2_) is within the range of [-1  1]. So the control parameter *r*_1_ plays a crucial role in the global exploration, which controls the transition of the algorithm from global exploration to local development. In the basic algorithm, the control parameter *r*_1_ adopts the linear decreasing method of (2) to guide the algorithm transit from the global exploration to the local development.

(2) $$\begin{array}{c} r_{1}=a\left(\;1-\frac{t}{N\_iter}\;\right) \end{array}$$

where *a* is a constant, *t* is the current iteration, and N_iter is the maximum number of iterations.

## 3. Modified Sine-Cosine Algorithm

### 3.1. Exponential Decreasing Conversion Parameter

The parameter setting is crucial to the search performance in the basic sine-cosine algorithm, in which the control parameter *r*_1_ controls the transition of algorithm from global exploration to local development. The larger value *r*_1_ can improve the global searching ability of the algorithm, and the smaller value *r*_1_can enhance the local development ability of the algorithm. Therefore, *r*_1_ is designed as the linear decreasing method of (2) in the basic algorithm to balance the global exploration and local development ability of the algorithm. In the literature [31], experimental contrast analysis is made on the linear decreasing method, parabola decreasing method, and exponential decreasing method in the basic algorithm. It is found that the exponential decreasing method is superior to the other two methods in the search performance. At the same time, the inertia weight remains unchanged in the iterative process of the basic algorithm, which may easily cause the population individuals to oscillate in the later period of search. In this paper, both the linear decreasing inertia weight and exponential decreasing conversion parameter strategy are used on the basis of (1), which can better balance the global exploration and local development ability of the algorithm. The update mode of individuals is as follows:

(3) pi,jt+1=ωt·pi,jt+r1·sin⁡r2·r3·pbest,jt−pi,jt,r4<0.5ωt·pi,jt+r1·cos⁡r2·r3·pbest,jt−pi,jt,r4≥0.5

(4) ωt=ωmax−ωmax−ωmin·tN_iter

(5) r1t=a·et/N_iter

where* t* is the current iteration, N_iter is the maximum number of iterations, *p*_*best*,*j*_^*t*^ is the* j*th dimension value of the optimal individual at iteration* t*, *p*_*i*,*j*_^*t*^ is the* j*th dimension value of the individual* i* of current iteration, and *ω*_max_ and *ω*_min_ are the maximum and minimum inertia weight, respectively.

It can be seen from (3) that the population individuals work together through the inertia weight *ω*(*t*) and conversion parameter *r*_1_(*t*). The value of *ω*(*t*) and *r*_1_(*t*) is large in the early iterations, which is conducive to the global exploration of the algorithm. The values of *ω*(*t*) and *r*_1_(*t*) are small in later iterations, which are conducive to the local development of the algorithm so as to improve the searching precision and convergence speed of the algorithm.

### 3.2. The Neighborhood Search of the Optimal Individual

In the basic sine-cosine algorithm, the search directions of the new individuals simply are updating process by optimal individuals in the population. Once the global optimal individuals fall into the local optimum, the whole algorithm easily gets into premature convergence. Therefore, in order to reduce the possibility of algorithm getting into the local optimum, the guiding role of the better individuals possibly existing near the optimal solution should be used. In this paper, the random individuals near the optimal solution are used to replace the current optimal individuals to guide the algorithm search, so as to improve the possibility of algorithm jumping out of the local optimum. The sine-cosine algorithm strategy for the neighborhood search of the optimal individual is

(6) $$\begin{array}{c} p_{i,j}^{t+1}=\left\{\begin{array}{cc} \omega \left(t\right)\cdot p_{i,j}^{t}+r_{1}\cdot \sin\left(r_{2}\right)\cdot \left|r_{3}\cdot p_{best,j}^{t}\cdot \left(1+\lambda \cdot unifrnd\left(-1,1\right)\right)-p_{i,j}^{t}\right|, & r_{4}<0.5\\ \omega \left(t\right)\cdot p_{i,j}^{t}+r_{1}\cdot \cos\left(r_{2}\right)\cdot \left|r_{3}\cdot p_{best,j}^{t}\cdot \left(1+\lambda \cdot unifrnd\left(-1,1\right)\right)-p_{i,j}^{t}\right|, & r_{4}\geq 0.5 \end{array}\right. \end{array}$$

where *unifrnd*(−1,1) is the uniform distribution number within (-1, 1), and *λ* is the disturbance coefficient. Other parameters are in line with (3).

In the neighborhood search of the optimal individual, the current optimal individual is taken as the center and *λ* as the step size, and the algorithm searches between the section *r*_3_ · *p*_*best*,*j*_^*t*^ · (1 − *λ* · *unifrnd*(0,1)) and *r*_3_ · *p*_*best*,*j*_^*t*^ · (1 + *λ* · *unifrnd*(0,1)). It effectively expands the search orientation and increases the probability of algorithm jumping out of the local optimum.

### 3.3. Greedy Levy Mutation

In the basic sine-cosine algorithm, the optimal individuals lead the search direction of the whole population. But the optimal individuals lack experiential knowledge and self-learning ability. So they may hardly get effective improvement and thus get into the domain of local optimum. In order to further prevent the basic sine-cosine algorithm from getting into the local optimum and eliminate the defect of low efficiency in later period, a strategy based on greedy Levy mutation is proposed for the optimum individuals. Thus, the population individuals can jump out of the position of optimal value searched previously through the mutation operation, which retains the diversity of population. The mutation method is as follows:

(7) $$\begin{array}{c} p_{best,j}^{t+1}=p_{best,j}^{t}+\theta \left(\;j\;\right)\cdot levy\cdot p_{best,j}^{t} \end{array}$$

where *levy* is the random number that obeys the Levy distribution, *θ*(*j*) is the coefficient of self-adapting variation, and *p*_*best*,*j*_^*t*^ is the* j*th dimension value of the optimal individual at iteration* t* (Algorithm 1).

#### 3.3.1. Random Number Generated According to the Levy Distribution

The *levy* flight is characterized by long-term short-distance migration and occasional long-distance jump, which is suitable for describing the life active law of many colonial organisms. In this paper, the characteristic of *levy* flight is used to form a *levy* mutation mechanism. This mechanism ensures that the proposed algorithm makes sufficient search near the area of the optimal individuals and has a certain mutation at the same time, which can improve the global searching ability of the algorithm. As the integral of probability density function of  *levy* distribution is difficult, it has been proved that Mantegna algorithm can be used to achieve the equivalent calculation [32]. That is,

(8) levy≈uv1/βu~N0,σu2,v~N0,σv2

where *σ*_*v*_ = 1, *β* = 3/2, and *σ*_*u*_ can be calculated based on

(9) $$\begin{array}{c} \sigma_{u}={\left\{\;\frac{\Gamma \left(1+\beta \right)\cdot sin\left(\pi \beta /2\right)}{\Gamma \left[\left(1+\beta \right)/2\right]\cdot \beta \cdot 2^{\left(\beta -1\right)/2}}\;\right\}}^{1/\beta } \end{array}$$

where Γ is the standard Gamma function.

#### 3.3.2. Coefficient of Self-Adapting Variation

The swarm intelligence optimization algorithm is generally divided into two stages in the iterative process, namely, global exploration at the earlier stage and local development at the later stage. Therefore, in order to achieve the goal of obtaining a big variation to conduct the global disturbances at the earlier stage and decreasing the variation range to accelerate the local search at the later stage, the proposed algorithm is used a self-adapting mutation strategy. The self-adapting variation control coefficient is in

(10) θj=e−ε·t/N_iter1−rj/rmaxj

(11) rj=pbest,jt−1n∑i=1npi,jt

(12) rmaxj=max⁡p:,jt−min⁡p:,jt

where* t* is the current iteration, N_iter is the maximum iteration, *ε* is the coefficient, *r*(*j*) is the difference between the* j*th dimension value of the current optimal individual and the* j*th dimension average value of the population individual, and *r*_max_(*j*)is the maximum distance of the* j*th dimension in the population.

From (10) ~ (12), it can be seen that the coefficient *θ*(*j*) can be mainly considered from both iterative process and diversity. The iterative part is controlled by the part of −*ε* · *t*/*N*_*iter*, and the diversity is adjusted by the part of 1 − *r*(*j*)/*r*_max_(*j*). On the early iterations, the individuals have poor performance and large diversity. So large coefficient can cause enough disturbances to the population and enhance the global searching ability. As iterations go on, the individuals in the population have better performance and gradually decrease coefficient, which can ensure that the algorithm converges to the optimal value smoothly to reduce the search oscillation of the optimal value. The solution method is shown in Algorithm 2.

### 3.4. The Modified Sine-Cosine Algorithm Based on the Greedy Levy Variation

The procedure of the improved sine-cosine algorithm based on neighborhood search and the greedy Levy variation is shown in Algorithm 3.

For the basic SCA algorithm, the time complexity of creating the initial population is *O*(*n*), the time complexity of performing sine and cosine operations is *O*(*n*_*iter∗n∗d*), and the cross-border processing is *O*(*n*_*iter∗n*). So the time complexity of the basic SCA algorithm is *O*(*n*)+*O*(*n*_*iter∗n*) + *O*(*n*_*iter∗n∗d*). In the MSCA algorithm, the time complexity of creating the initial population is *O*(*n*), and the time complexity of calculating *ϖ*(*t*) and *r*_1_ is *O*(2*∗n*_*iter*). The time complexity of performing the sine and cosine operations based on the neighborhood search is *O*(*n*_*iter∗n∗d*). The time complexity of cross-border processing is *O*(*n*_*iter∗n*), and the time complexity of the greedy Levy mutation operation is *O*(*n*_*iter∗d∗n*). Therefore, the time complexity of the MSCA algorithm is *O*(*n*) + *O*(2*∗n*_*iter*) + *O*(*n*_*iter∗n∗d*) + *O*(*n*_*iter∗n*) + *O*(*n*_*iter∗d∗n*) = *O*(*n*) + *O*((*n* + 2)*∗n*_*iter*) + *O*(2*∗n*_*iter∗d∗n*). Obviously, the time complexity of the MSCA algorithm is higher than that of the standard SCA algorithm while both of them are in the same order of magnitude.

## 4. Experimental Simulation

In order to verify the performance of MSCA, the experiment will be conducted from the following three aspects: (1) Contrast experiment is conducted between MSCA and particle swarm optimization (PSO) [8], differential evolution (DE) [9], bat algorithm (BA) [33, 34], teaching-learning-based optimization (TLBO) [35, 36], grey wolf optimizer (GWO) [37], and basic SCA algorithm. (2) The effectiveness of 3 improvement strategies is analyzed. (3) The parameter *λ* in the optimal individual neighborhood search strategy and parameter *ε* in the greedy *levy* mutation strategy are analyzed, respectively, and the effectiveness of the algorithm is discussed, so that the specific reference value of the above parameters in the algorithm can be determined.

### 4.1. Test Function and Experimental Platform

#### 4.1.1. Experimental Platform

In order to provide a comprehensive and full test environment, the simulation experiment is conducted in the test environment with operating system of Windows 10, CPU of Intel (R) Core (TM) i5-4210U (quad core), dominant frequency of 2.4GHZ and internal storage of 4GB, and programming tool of Matlab 2016b.

#### 4.1.2. Benchmark Functions

In order to validate the performance of the presented algorithm, 20 benchmark test functions in the literature [38, 39] are selected as experimental subjects, which have been widely used in the test. The benchmark test functions selected can be categorized into three types (i.e., unimodal high-dimensional functions, multimodal high-dimensional functions, and multimodal low-dimensional functions ). *f*_1_ ~ *f*_7_ are the unimodal high-dimensional functions, and they can be used to investigate the optimization precision of the algorithm, which can hardly converge to the global optimal point. *f*_8_ ~ *f*_13_ are the multimodal high-dimensional functions with several local extreme points, which can be used to test the global searching performance and ability to avoid premature convergence of the algorithm. *f*_14_ ~ *f*_20_ are the multimodal low-dimensional functions. As the optimal value of the most test functions is zero, we select some test functions with nonzero optimal value. The function name, expression, dimension, search range, and theoretical optimal value are shown in Table 1.

### 4.2. Contrastive Analysis of Sine-Cosine Algorithm Based on Greedy Levy Mutation

In order to evaluate the performance of the algorithm proposed in this paper, six algorithms are selected as contrast algorithms in the experiment, that is, PSO, DE, BA, TLBO, GWO, and SCA, respectively. The contrast algorithms selected the same parameters as the original literature and the parameter setting as shown in Table 2. The parameters of the MSCA algorithm are set as follows. The population size is 100. The minimum inertia weight *ω*_max_ is 0.9. The minimum inertia weight *ω*_min_ is 0.4. *ε* is 30. *λ* is 0.01. The other parameters are consistent with the basic SCA. For each test function, the number of iterations is 5000, and each algorithm runs independently 20 times. The performance of each algorithm is measured by four indexes, which are optimal value, average value, worst value, and variance. The statistical results are as shown in Tables 3–5.

It can be seen from Table 3 that 5 theoretical optimal values (*f*_1_,*f*_2_,*f*_3_,*f*_4_, and*f*_6_) are searched by the MSCA algorithm for the 7 unimodal high-dimensional functions, and the searching precision of another two functions (*f*_5_ and *f*_7_) is also close to the theoretical optimal values. The MSCA algorithm performs better than PSO, DE, BA, and CSA algorithms in the aspect of optimal value, average value, worst value, and variance. For *f*_1_, *f*_2_, and*f*_4_, both TLBO algorithm and MSCA algorithm can search the global optimal theoretical value. For *f*_3_, *f*_5_, *f*_6_, and *f*_7_, the MSCA algorithm obtains better results than the TLBO algorithm. The MSCA algorithm obtains better results than the GWO algorithm besides *f*_1_ (both algorithms can search the global optimal value). It shows that the MSCA algorithm has a great advantage in the searching precision of unimodal high-dimensional problems.

From the search results of the multimodal high-dimensional functions in Table 4, it can be seen that 3 functions (*f*_8_, *f*_9_, and *f*_11_) obtain the globally optimal solution in the MSCA algorithm, and the search results of the other functions are also better than in the other 6 algorithms. The search result of the PSO algorithm is not good, and the search result of the DE algorithm is better than BA, TLBO, GWO, and CSA algorithms. In contrast to TLBO, MCSA has better performance in the aspect of optimal value, average value, worst value, and variance (besides*f*_11_), which indicates the superiority of optimization results of the MSCA in the multimodal high-dimensional functions.

It can be seen from Tables 3 and 4 that the search ability of MCSA is better than that of the TLBA in most high-dimensional functions. Both MCSA and TLBA find out the global optimizing in other functions (i.e., *f*_1_, *f*_2_, *f*_4_, and *f*_11_).

For multimodal low-dimensional functions (*f*_14_ ~ *f*_20_), most functions have the characteristics of strong shocks. The low-dimensional functions are usually used to test the ability of the algorithm in breaking away from the local optimum. From the search results of low-dimensional multimodal functions in Table 5, it can be seen that the MSCA algorithm obtains the global optimal solution of all functions, while the basic CSA algorithm has poor stability in solving such problems. MSCA, DE, TLBO, and GWO can obtain theoretical optimal value, illustrating that the four algorithms have the ability of jumping out the local optimal values in multimodal low-dimensional functions.

**Figure 1:**
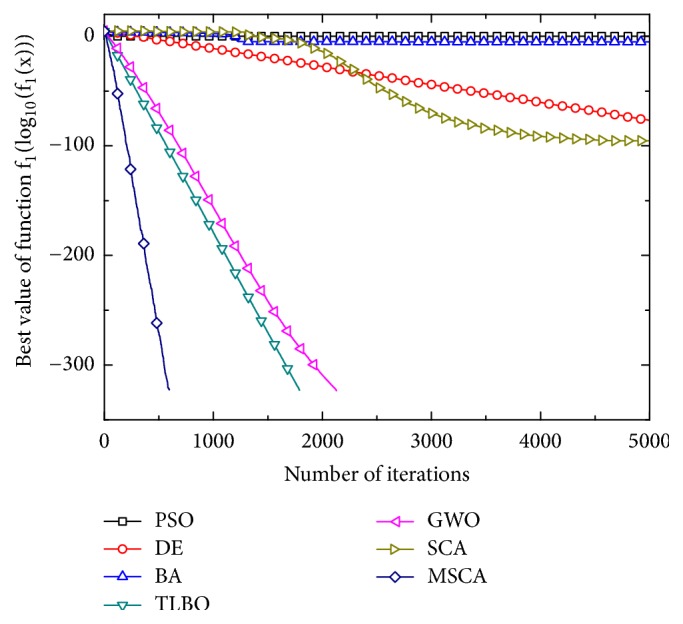
Convergence rates for *f*_1_(*x*).

**Figure 2:**
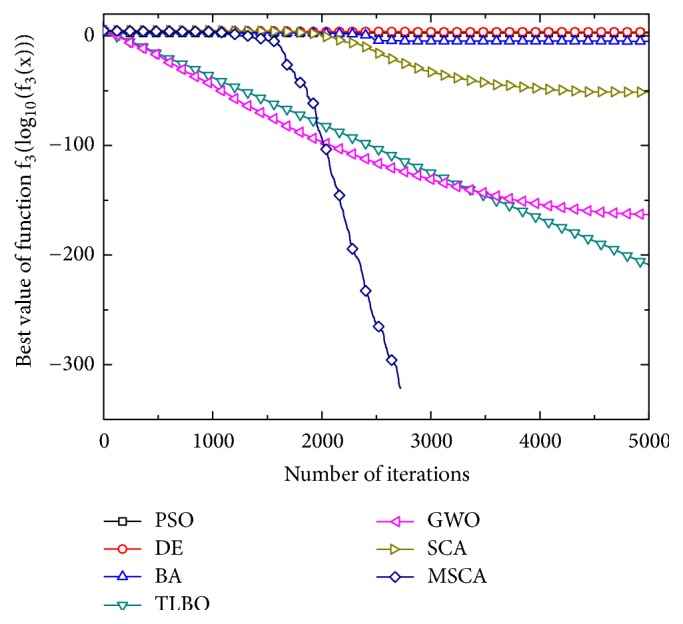
Convergence rates for *f*_3_(*x*).

**Figure 3:**
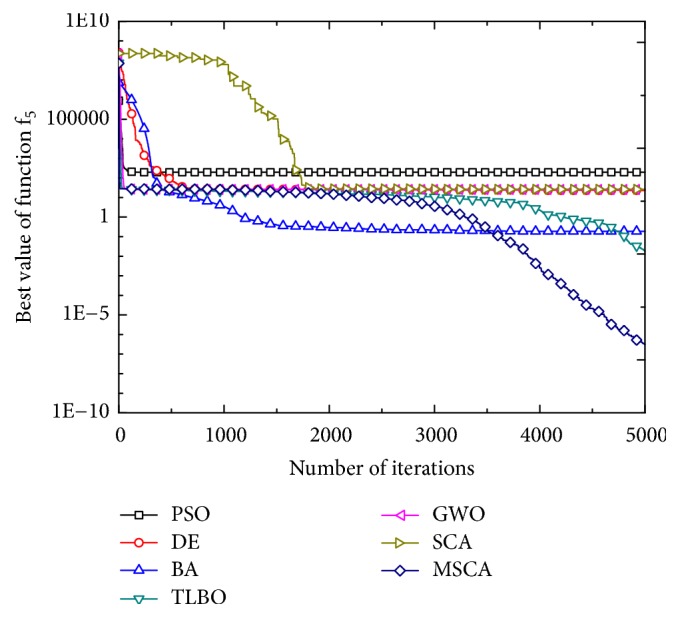
Convergence rates for *f*_5_(*x*).

**Figure 4:**
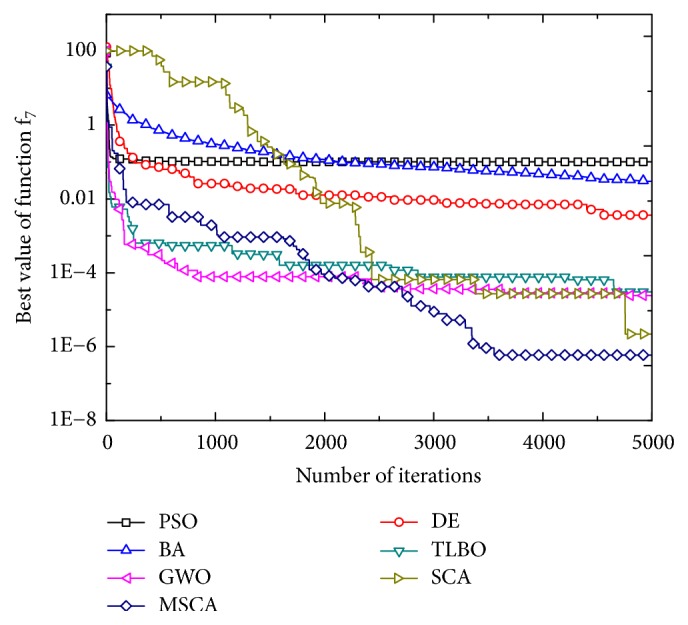
Convergence rates for *f*_7_(*x*).

**Figure 5:**
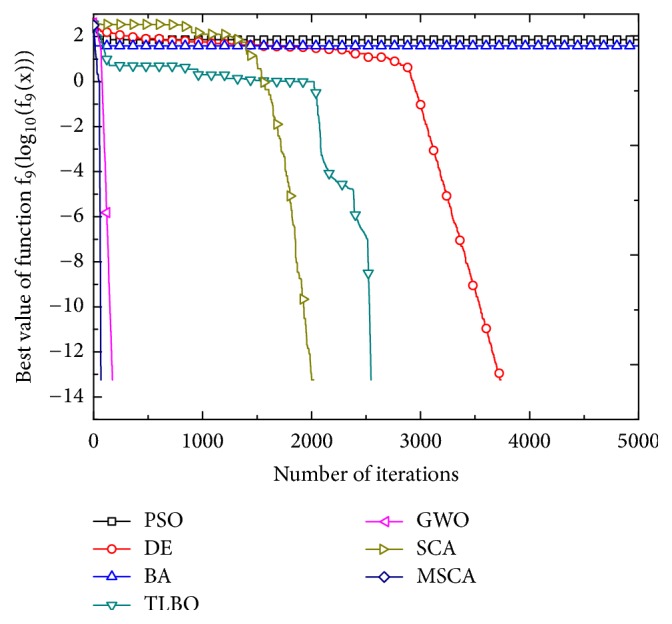
Convergence rates for *f*_9_(*x*).

**Figure 6:**
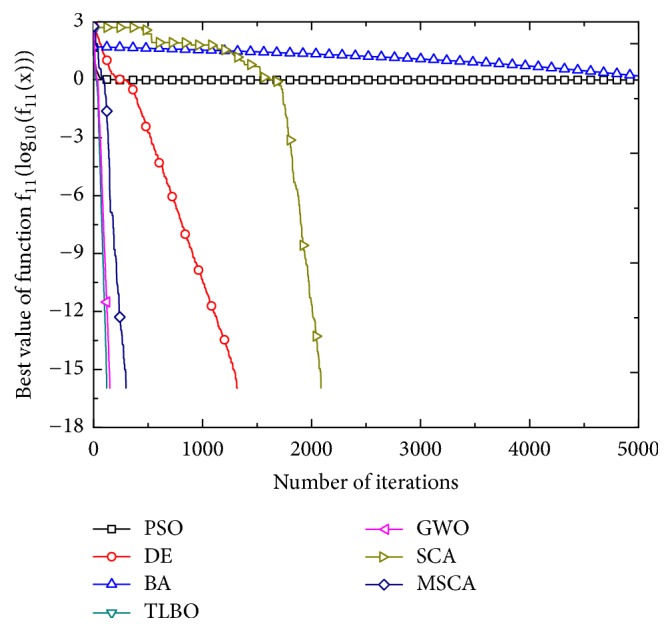
Convergence rates for *f*_11_(*x*).

**Figure 7:**
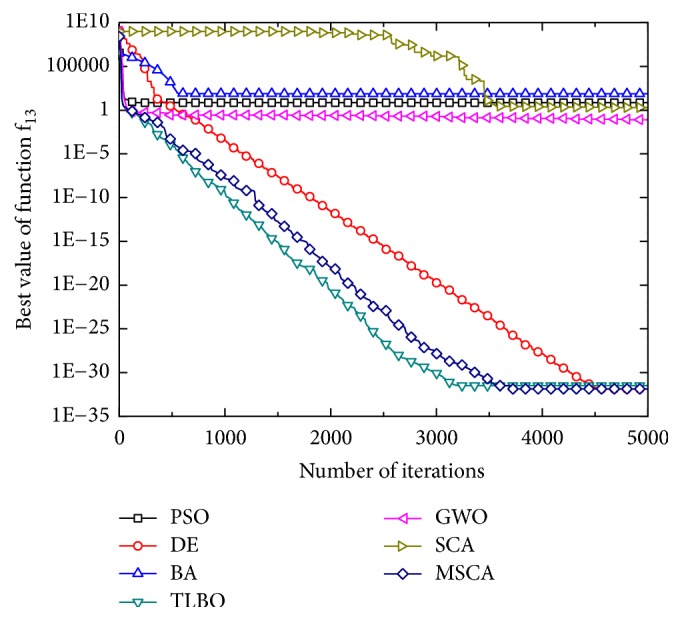
Convergence rates for *f*_13_(*x*).

Figures 1–7 show the convergence curves of optimal results for some high-dimensional functions by the 7 algorithms. The data in the figures show the optimal results based on the 7 algorithms after 20 independent experiments. For the convenience of drawing, the abscissa takes the number of iterations, and the ordinate takes the logarithm of fitness value for *f*_1_, *f*_3_, *f*_9_, and *f*_11_. Besides, the ordinate takes the fitness value for *f*_5_, *f*_7_, and *f*_13_. It can be seen from Figures 1–7 that the MSCA algorithm has faster convergence speed and higher optimization precision than the other 6 intelligence algorithms.

In order to verify that the performance of the proposed algorithm has significant advantages over other intelligence algorithms, the statistics are carried out (optimal value, average value, worst value, and variance) for the 7 algorithms after 20 independent experiments, and t-test is also used in the experiments for the significance analysis of the optimization results. The function* ttest (x,y,0.05, “left”)* is verified in the experiments. Here, “x” means the experimental result of MSCA algorithm. “y” means the experimental result of contrast algorithms. The significance level is 0.05, and “left” means left-tailed test. The test results are shown in Table 6. “+” indicates that the MSCA algorithm has significant advantages over the contrast algorithms. “≈” indicates that there is no significant difference between the MSCA algorithm and the contrast algorithms. “-” indicates that the MSCA algorithm is inferior to the contrast algorithms. According to the data listed in correlation Table 6, compared with the PSO, DE, BA, TLBO, GWO, and SCA algorithms, there are 20, 13, 19, 12, 15, and 17 test functions, respectively, in significant advantages. For *f*_18_, the search results of the MSCA algorithm are inferior to that of the DE, BA, and TLBO algorithms. In addition, there is no significant difference between the MSCA algorithm and other contrast algorithms for the search results of other test functions (such as*f*_6_, *f*_9_, *f*_11_, *f*_13_, *f*_14_, *f*_15_, and*f*_16_ in the DE algorithm). The main reason is that both the MSCA algorithm and contrast algorithms can obtain the global theoretical solution.

### 4.3. Efficiency Analysis of the Improvement Strategy

In order to analyze the influence of the three improvement strategies on the performance of SCA algorithm, the odd-numbered standard test functions in Table 1 have been used to experimentalize. In the C-SCA algorithm, the linear decreasing inertia weight and exponential decreasing conversion parameter strategy are combined with the basic SCA algorithm. In the N-SCA algorithm, optimal individual neighborhood search strategy is combined with the basic SCA algorithm. In the G-SCA algorithm, the greedy Levy mutation strategy is combined with the basic SCA algorithm. The C-SCA, N-SCA, G-SCA, and the basic SCA are compared with the proposed algorithm. The experimental parameters are consistent with those in Section 4.2.  Table 7 summarizes the experimental results of the three strategies and the proposed algorithm. It can be seen from the experimental results that the C-SCA which used a single strategy makes a limited improvement on the search performance of the functions besides *f*_1_(*x*) and *f*_3_(*x*). The N-SCA algorithm is basically the same as the basic SCA algorithm. The G-SCA strategy has better improvement effect on the test functions *f*_1_(*x*), *f*_3_(*x*), and *f*_7_(*x*), while it has basically the same search results of other test functions as the basic SCA algorithm. However, when the three improvement strategies work together with the SCA algorithm, the search performance of the proposed algorithm can be greatly improved. The main reasons are analyzed as follows. Firstly, the optimal individual neighborhood research allows the random individuals near the current optimal individuals to play the roles of the leader, which increases the probability of the proposed algorithm jumping out of the local optimal solution. Secondly, the greedy Levy mutation strategy increases the diversity of population and adequacy of local search. Thirdly, as the linear decreasing inertia weight and exponential declining conversion parameter method are used, the algorithm chooses larger inertia weight value and conversion parameter value in the early iteration, which is conducive to the global searching ability of the algorithm. In the later iteration, the algorithm chooses smaller values, which is conducive to the local search. Thus, the presented algorithm avoids falling into the local optimum. The solution precision and convergence speed are significantly improved by the collaboration of the three improvement strategies.

From the results of Wilcoxon rank sum test in Table 8, it can be seen that the C-SCA algorithm has significant advantages over the basic SCA algorithm only in the test results of functions *f*_1_(*x*), *f*_3_(*x*), and *f*_13_(*x*). There is no significant difference between the N-SCA algorithm and the basic SCA algorithm. The G-SCA algorithm has significant advantages over the basic SCA algorithm in the searching performance other than *f*_9_(*x*) and *f*_11_(*x*).

### 4.4. Parameter Sensitivity Analysis in the Algorithm

#### 4.4.1. The Analysis of Parameter *λ* in the Optimal Individual Domain Search Strategy

In order to explore the influence of the parameter *λ* in the optimal individual domain search strategy, the even-numbered standard test functions in Table 1 are selected. The parameter *λ* takes 0.005, 0.01, 0.02, 0.03, and 0.05, respectively, for independent experiments, with other parameters unchanged. The optimal individual domain search strategy independently acts on the SCA algorithm (N-SCA).  Table 9 summarizes the results when the N-SCA algorithm takes different values of *λ*. Here, the black boldface means the winners in the comparison expressed by “+”. It can be seen from the last row of Table 9 that the number of the winners is 3 when *λ* = 0.01, which is better than other cases. Therefore, *λ* = 0.01 is the optimal parameter selected.

#### 4.4.2. The Analysis of Parameter *ε* in the Greedy *levy* Mutation Strategy

The value of parameter *ε* has a great effect on the algorithm performance in the self-adapting mutation mode adopted in (10). In order to explore the influence of the parameter *ε* on the searching performance of the algorithm, the even-numbered standard test functions in Table 1 are selected. The parameter *ε* takes 10, 30, 60, and 90, respectively, for independent experiments, with other parameters unchanged. The greedy *levy* mutation strategy independently acts on the SCA algorithm (G-SCA).  Table 10 summarizes the results when the G-SCA algorithm takes different values of *ε*. Here, the optimal results are marked with “+” and showed by overstriking. It can be seen from Table 10 that when *ε* takes 10, 30, 60, and 90, respectively, the number of optimal search results obtained by GLM-SCA is 1, 5, 0, and 1, respectively. When *ε*=30, the search results of GLM-SCA are much better than those of other values. Therefore, *ε*=30 is a reasonable parameter chosen.

## 5. Conclusion

An improved sine-cosine algorithm based on greedy *levy* mutation is proposed in this paper. The proposed algorithm adopts the method of both exponential decreasing conversion parameter and linear decreasing inertia weight to better balance the global searching and local development ability of the algorithm. The update mode guided by the of random individual near the optimal individuals is introduced, which increases the probability of algorithm jumping out of the local extremum. Inspired by the *levy* flight mode of long-term short-distance migration and occasional long-distance jump, a self-adapting greedy *levy* mutation strategy is designed to mutate the optimal individuals. The proposed strategy can increase the population diversity and reduce the search oscillation of algorithm, making the algorithm converge to the global optimum smoothly. Twenty typical benchmark test functions are applied to verify the performance of the proposed algorithm. The results show that the searching precision and convergence speed of the proposed algorithm can be greatly improved through the collaboration of the three improvement strategies. At the same time, the contribution of the three improvement strategies to the proposed algorithm is analyzed in detail. The influence of parameter selection on the algorithm performance is discussed, and suggestions on parameter selection are also given in this paper. However, the proposed algorithm is still theoretically and practically in its infancy stage, and the setting of the parameters in the algorithm is determined by empirical tests. At the same time, when the algorithm introduces greedy Levy mutation strategy, the time complexity of the algorithm is greatly increased. Therefore, the proposed algorithm only conducts the greedy Levy mutation strategy on the best individual at each iteration.

## Figures and Tables

**Algorithm 1:**
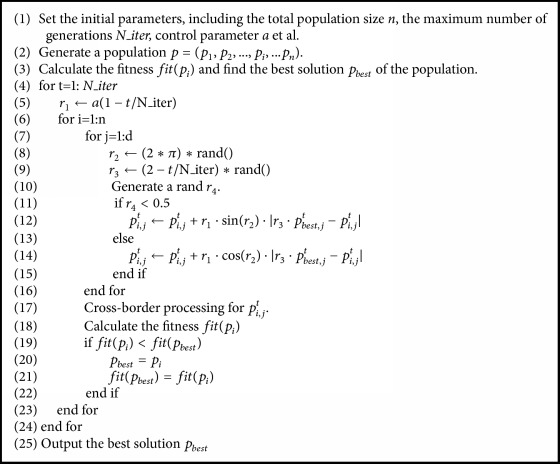
The pseudocode of the basic sine-cosine algorithm.

**Algorithm 2:**
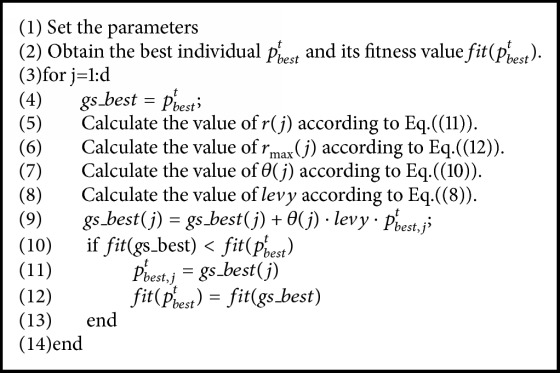
The pseudocode of the optimal individual based on greedy levy variation.

**Algorithm 3:**
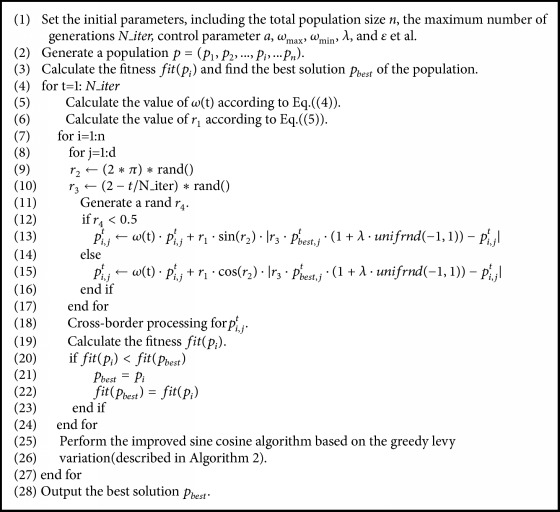
Algorithm 3 is the pseudocode of the improved sine-cosine algorithm based on the greedy levy variation.

**Table 1:** Standard test functions.

No	Name	Benchmark test functions	Dimension	Scope	Optimum
*f*_1_(*x*)	Sphere Model	$f(x)=\overset{D}{\underset{i=1}{\sum }}x_{i}^{2}$	30	[-100,100]	0

*f*_2_(*x*)	Schwefel's Problem 2.22	$f(x)=\overset{D}{\underset{i=1}{\sum }}|x_{i}|+\prod_{i=1}^{D}|x_{i}|$	30	[-10,10]	0

*f*_3_(*x*)	Schwefel's Problem 1.2	$f(x)=\overset{D}{\underset{i=1}{\sum }}{\left(\sum_{j=1}^{i}x_{i}\right)}^{2}$	30	[-100,100]	0

*f*_4_(*x*)	Schwefel's Problem 2.21	$f(x)=\overset{D}{\underset{i=1}{max}}\left\{\left|x_{i}\right|\right\}$	30	[-100,100]	0

*f*_5_(*x*)	Generalized Rosenbrock's Function	$f(x)=\overset{n-1}{\underset{i=1}{\sum }}\left[100{\left(x_{i+1}\text{-}x_{i}^{2}\right)}^{2}+{\left(1\text{-}x_{i}\right)}^{2}\right]$	30	[-30,30]	0

*f*_6_(*x*)	Step Function	$f(x)=\overset{D}{\underset{i=1}{\sum }}{\left(\left\lfloor x_{i}+0.5\right\rfloor \right)}^{2}$	30	[-100,100]	0

*f*_7_(*x*)	Quartic Function i.e. Noise	$f(x)=\overset{D}{\underset{i=1}{\sum }}i\cdot x_{i}^{4}+random(0,1)$	30	[-1.28,1.28]	0

*f*_8_(*x*)	Generalized Schwefel's Problem 2.26	$f(x)=\overset{D}{\underset{i=1}{\sum }}-x_{i}\cdot sin\left(\sqrt{\left|x_{i}\right|}\right)$	30	[-500,500]	-418.9829*∗*n

*f*_9_(*x*)	Generalized Rastrigin's Function	$f(x)=\overset{n}{\underset{i=1}{\sum }}(x_{i}^{2}-10\cdot cos(2\cdot \pi \cdot x_{i})+10)$	30	[-5.12,5.12]	0

*f*_10_(*x*)	Ackley's Function	$f(x)=-20exp\left(-0.2\sqrt{\frac{1}{d}\overset{d}{\underset{i=1}{\sum }}x_{i}^{2}}\right)-exp\left(\frac{1}{d}\overset{d}{\underset{i=1}{\sum }}\cos\left(2\pi x_{i}\right)\right)+20+e$	30	[-32,32]	0

*f*_11_(*x*)	Generalized Griewank Function	$f(x)=\frac{1}{4000}\cdot \overset{n}{\underset{i=1}{\sum }}x_{i}^{2}\text{-}\;\overset{n}{\underset{i=1}{\prod }}cos\frac{x_{i}}{\sqrt{i}}+1$	30	[-600,600]	0

*f*_12_(*x*)	Generalized Penalized Function	$\begin{array}{c} f(x)=\frac{\pi }{D}\left\{10sin^{2}(\pi \cdot y_{1})+\overset{D-1}{\underset{i=1}{\sum }}(y_{i}-1{)}^{2}[1+10sin^{2}(\pi \cdot y_{i+1})]+(y_{n}-1{)}^{2}\right\}\\ +\overset{n}{\underset{i=1}{\sum }}u(x_{i},10,100,4) \end{array}$	30	[-50,50]	0
		$y_{i}=1+\frac{x_{i}+1}{4}$			
		$u(x_{i},\alpha ,k,m)=\left\{\begin{array}{cc} k(x_{i}-\alpha {)}^{m}, & x_{i}>\alpha \\ 0, & -\alpha \leq x_{i}\leq \alpha \\ k(-x_{i}-\alpha {)}^{m}, & x_{i}\leq \alpha \end{array}\right.$			

*f*_13_(*x*)	Generalized Penalized Function	$\begin{array}{c} f(x)=0.1\left\{10sin^{2}(3\pi \cdot x_{1})+\overset{D-1}{\underset{i=1}{\sum }}(x_{i}-1{)}^{2}[1+10sin^{2}(3\pi \cdot x_{i+1})]+(x_{n}-1{)}^{2}\right\}\\ +\overset{n}{\underset{i=1}{\sum }}u(x_{i},10,100,4) \end{array}$	30	[-50,50]	0

*f*_14_(*x*)	Shekel's Foxholes Function	$f(x)={\left[\frac{1}{500}+\sum_{j=25}^{25}\frac{1}{j+\sum_{i=1}^{2}{\left(x_{i}-a_{ij}\right)}^{6}}\right]}^{-1}$	2	[-65.56,65.56]	0.9980

*f*_15_(*x*)	Kowalik's Function	$f(x)={\sum_{i=1}^{11}\left[a_{i}-\frac{x_{1}(b_{i}^{2}+b_{i}x_{2})}{b_{i}^{2}+b_{i}x_{3}+x_{4}}\right]}^{2}$	4	[-5,5]	0.0003075

*f*_16_(*x*)	Six‐Hump Camel‐Back Function	$f\left(x\right)=4x_{1}^{2}-2.1x_{1}^{4}+\frac{x_{1}^{6}}{3}+x_{1}x_{2}-4x_{2}^{2}-4x_{2}^{4}$	2	[-5,5]	-1.0316285

*f*_17_(*x*)	Branin Function	$f(x)={\left(x_{2}-\frac{5.1}{4\pi^{2}}x_{1}^{2}+\frac{5}{\pi }x_{1}-6\right)}^{2}+10\left(1-\frac{1}{8\pi }\right)cosx_{1}+10$	2	[-5,10; 0,15]	0.398

*f*_18_(*x*)	Goldstein‐Price Function	$\begin{array}{c} f(x)=[1+(x_{1}+x_{2}+1{)}^{2}(19-14x_{1}+3x_{1}^{2}-14x_{2}+6x_{1}x_{2}+3x_{2}^{2})]\\ \left[30+(2x_{1}-3x_{2}{)}^{2}(18-32x_{1}+12x_{1}^{2}+48x_{2}-36x_{1}x_{2}+27x_{2}^{2})\right] \end{array}$	2	[-2,2]	3

*f*_19_(*x*)	Hartman's Function	$f(x)=-\overset{4}{\underset{i=1}{\sum }}c_{i}\cdot exp\left[-\overset{3}{\underset{j=1}{\sum }}a_{ij}(x_{j}-p_{ij}{)}^{2}\right]$	3	[0,1]	-3.8628

*f*_20_(*x*)	Hartman's Function	$f(x)=-\overset{4}{\underset{i=1}{\sum }}c_{i}\cdot exp\left[-\overset{6}{\underset{j=1}{\sum }}a_{ij}(x_{j}-p_{ij}{)}^{2}\right]$	6	[0,1]	-3.32

**Table 2:** The parameters set of all other algorithms.

Algorithms	Parameters
PSO	the population size is 100, c1 = 1.49445, c2 = 1.49445, *ϖ* = 0.729
DE	the population size is 100, pCR=0.2, *β*_min_ = 0.2, *β*_max_ = 0.8
BA	the population size is 100, Qmin=0, Qmax=2, *R*^0^ = 0.1,*A* = 0.9,*α* = 0.95,*γ* = 0.9
TLBO	the population size is 100, TF=2 or 1
GWO	the population size is 100
SCA	the population size is 100, a=2

**Table 3:** Test statistical results of functions *f*_1_ ~ *f*_7_.

Benchmark function		PSO	DE	BA	TLBO	GWO	CSA	MSCA
*f*_1_(*x*)	Best	9.07650779E-01	1.65407264E-77	6.60911194E-06	0	0	2.41227653E-99	0
	Mean	1.97056172E+01	2.62600749E-76	8.47162999E-06	0	0	7.79692173E-86	0
	Worst	4.94364998E+01	6.31133690E-76	1.067648634E-05	0	0	1.54558620E-84	0
	Std	1.21250926E+01	2.65771348E-76	1.08812519E-06	0	0	3.45449900E-85	0

*f*_2_(*x*)	Best	7.49555834E+00	4.70179283E-46	1.03934630E-02	0	1.33605985E-252	2.60844948E-63	0
	Mean	1.12174418E+01	7.29820866E-46	3.22204665E+01	0	1.92526708E-251	2.41170865E-57	0
	Worst	2.02022159E+01	1.09423091E-45	9.30092920E+01	0	4.70878320E-251	4.40324480E-56	0
	Std	3.24260462E+00	2.84907171E-46	4.25000382E+01	0	0.00000000E+00	9.80901847E-57	0

*f*_3_(*x*)	Best	4.98125395E+02	3.62807984E+03	9.12788809E-06	3.04791020E-210	1.41705680E-163	4.35895925E-52	0
	Mean	1.26469111E+03	7.46153322E+03	1.50873736E-05	1.59616421E-203	1.49297576E-147	4.66854445E-46	0
	Worst	2.34229374E+03	1.07658866E+04	2.06438516E-05	1.07878223E-202	7.46124358E-147	4.16089002E-45	0
	Std	5.55711673E+02	2.67341362E+03	3.88507836E-06	0	3.33636341E-147	1.08000440E-45	0

*f*_4_(*x*)	Best	6.02122506E+00	3.83820416E-07	9.12788809E-06	0	3.88068837E-114	3.39775522E-34	0
	Mean	8.61122109E+00	5.60317284E-07	1.50873736E-05	0	1.33829026E-112	4.65163872E-26	0
	Worst	1.21184255E+01	7.68100603E-07	2.06438516E-05	0	5.28812647E-112	9.30221952E-25	0
	Std	1.92051215E+00	1.68313586E-07	3.88507836E-06	0	2.22979531E-112	2.08002707E-25	0

*f*_5_(*x*)	Best	1.98124514E+02	2.30707248E+01	1.86517166E-01	1.78074032E-02	2.51787920E+01	2.62363000E+01	7.76116677E-12
	Mean	1.01254587E+03	3.61624708E+01	1.79820932E+00	1.16810392E+00	2.61197789E+01	2.70812117E+01	5.82095129E-06
	Worst	3.26561071E+03	8.05070422E+01	4.19411715E+00	4.16880641E+00	2.70635443E+01	2.86501385E+01	2.47408894E-05
	Std	9.62804586E+02	2.48173576E+01	2.18569786E+00	1.09797628E+00	9.14735948E-01	6.82466657E-01	6.12299693E-11

*f*_6_(*x*)	Best	9.25831394E+00	0.00000000E+00	7.20895352E-06	3.69778549E-32	2.60632647E-07	3.37812908E+00	0
	Mean	2.87969807E+01	0.00000000E+00	7.97379259E-06	3.12154725E-31	1.00333382E-01	3.73341278E+00	0
	Worst	6.59256512E+01	0.00000000E+00	8.81647389E-06	1.35893617E-30	2.51573695E-01	4.00431209E+00	0
	Std	1.56322191E+01	0.00000000E+00	6.01088500E-07	3.40671290E-31	1.37387749E-01	1.64786441E-01	0

*f*_7_(*x*)	Best	1.01845349E-01	3.67133207E-03	2.30691046E-02	3.05530711E-05	2.42052731E-05	2.03984969E-06	5.95321712E-07
	Mean	2.09157287E-01	4.53974219E-03	3.06138991E-02	8.56177810E-05	5.18444251E-05	1.75661500E-05	2.06970501E-05
	Worst	3.76016628E-01	5.50134798E-03	3.63388054E-02	1.35008643E-04	9.24177878E-05	5.33965433E-05	5.50713991E-05
	Std	8.23611787E-02	6.65597117E-04	4.92468230E-03	2.77562650E-05	2.84763595E-05	1.42228474E-05	1.31769905E-05

**Table 4:** Test statistical results of functions *f*_8_ ~ *f*_13_.

Benchmark function		PSO	DE	BA	TLBO	GWO	CSA	MSCA
*f*_8_(*x*)	Best	-8.78252861E+3	-12569.48662	-8046.935903	-9477.60	-6861.79	-5025.22	-12569.5
	Mean	-6.77371061E+3	-12569.48662	-7449.534539	-8504.46	-6279.36	-4343.67	-12569.5
	Worst	-5.28468708E+3	-12569.48662	-6546.887655	-7219.72	-5690.82	-3996.64	-12569.5
	Std	8.52018857E+2	0	6.26E+02	6.27E+02	4.86E+02	2.59E+02	4.46E-09

*f*_9_(*x*)	Best	7.14247120E+1	0	3.88050502E+1	0.00000000E+00	0	0	0
	Mean	1.04062178E+2	0	6.88525758E+1	1.10328088E+01	0	0	0
	Worst	1.39959354E+2	0	9.25326145E+1	1.89042170E+01	0	0	0
	Std	1.67952026E+1	0	2.00896972E+1	5.03185256E+00	0	0	0

*f*_10_(*x*)	Best	7.14247120E+1	7.99360578E-15	1.27436962E+1	4.44089210E-15	4.44089210E-15	4.44089210E-15	8.88178420E-16
	Mean	1.04062178E+2	7.99360578E-15	1.40994295E+1	6.03961325E-15	6.57252031E-15	5.11344290E-01	8.88178420E-16
	Worst	1.39959354E+2	7.99360578E-15	1.52236570E+1	7.99360578E-15	7.99360577E-15	6.42084900	8.88178420E-16
	Std	1.67952026E+1	0	1.00814552	1.81336825E-15	1.94590142E-15	1.47695842	0

*f*_11_(*x*)	Best	9.88503548E-01	0	1.58288061	0	0	0	0
	Mean	1.14979639	0	5.23743048	0	0	0	0
	Worst	1.52716622	0	8.12540112	0	0	0	0
	Std	1.32482422E-01	0	2.85874482	0	0	0	0

*f*_12_(*x*)	Best	1.63117596	1.57054477E-32	5.12364688E-8	1.74802573E-32	6.59326073E-03	2.39498372E-01	1.57054477E-32
	Mean	5.10089444	1.57054477E-32	9.18135651E-1	4.42045801E-31	1.82811094E-02	3.34723047E-01	1.57094814E-32
	Worst	1.15654267E+01	1.57054477E-32	3.65772525	2.24517006E-30	3.24795465E-02	4.82802812E-01	1.57861209E-32
	Std	2.83503379	0	1.53913291	7.59973303E-31	9.60865202E-03	4.90236201E-02	1.80390681E-35

*f*_13_(*x*)	Best	6.87542849	1.34978380E-32	6.72918021E+1	3.32193607E-32	4.22190070E-07	1.82820415	1.34978380E-32
	Mean	2.47313999E+01	1.34978380E-32	7.67009096E+1	2.33370756E-02	2.37062320E-01	2.03922706	1.34978380E-32
	Worst	5.64417701E+01	1.34978380E-32	9.56157748E+1	1.41320020E-01	4.97953608E-01	2.22177498	1.34978380E-32
	Std	1.57138147E+01	0	1.14084239E+1	3.71532141E-02	2.05206244E-01	1.06729486E-01	2.80801150E-48

**Table 5:** Test statistical results of functions *f*_14_ ~ *f*_20_.

Benchmark function		PSO	DE	BA	TLBO	GWO	CSA	MSCA
*f*_14_(*x*)	Best	0.99800384	0.99800384	0.99800384	0.99800384	0.99800384	0.99800384	0.998003838
	Mean	1.29561904	0.99800384	3.36874514	0.99800384	0.99800384	0.99800387	0.998003838
	Worst	2.98210516	0.99800384	6.90333569	0.99800384	0.99800384	0.99800414	0.998003838
	Std	0.72687065	0	2.26483904	0	0	0.00000007	0

*f*_15_(*x*)	Best	0.00030752	0.00030749	0.00030749	0.00030749	0.00030749	0.00031054	0.00030748598
	Mean	0.00347700	0.00030749	0.00030749	0.000307486	0.00049062	0.00041204	0.00030748598
	Worst	0.02036334	0.00030749	0.00030749	0.000307486	0.00122317	0.00126659	0.00030748598
	Std	0.00694358	0	0	1.50786E-19	0.00040951	0.00029067	0

*f*_16_(*x*)	Best	-1.03162845	-1.03162845	-1.03162845	-1.03162845	-1.03162845	-1.03162842	-1.03162845
	Mean	-1.03162842	-1.03162845	-1.03162845	-1.03162845	-1.03162845	-1.03162684	-1.03162845
	Worst	-1.03162833	-1.03162845	-1.03162845	-1.03162845	-1.03162845	-1.03162424	-1.03162845
	Std	0.00000004	0	0	2.27813E-16	0	0.00000122	0

*f*_17_(*x*)	Best	0.39788737	0.397887358	0.39788736	0.397887358	0.39788736	0.39788838	0.397887358
	Mean	0.39788774	0.397887358	0.39788736	0.397887358	0.39789181	0.39799018	0.397887358
	Worst	0.39788882	0.397887358	0.39788736	0.397887358	0.39790961	0.39837908	0.397887358
	Std	0.00000041	0	0	0	0.00000995	0.00013970	0

*f*_18_(*x*)	Best	3.00000000	3.00000000	3.00000000	3.00000000	3.00000000	3.00000000	3.00000000
	Mean	3.00000553	3.00000000	3.00000000	3.00000000	3.00000006	3.00000003	3.00000000
	Worst	3.00002104	3.00000000	3.00000000	3.00000000	3.00000010	3.00000008	3.00000000
	Std	0.00000656	0	0	7.62408E-16	0.00000004	0.00000003	0

*f*_19_(*x*)	Best	-3.86278215	-3.86278215	-3.86278214	-3.86278215	-3.86278215	-3.86226093	-3.86278215
	Mean	-3.86278211	-3.86278215	-3.86278214	-3.86278215	-3.86278203	-3.85557758	-3.86278215
	Worst	-3.86278193	-3.86278215	-3.86278213	-3.86278215	-3.86278159	-3.85462395	-3.86278215
	Std	0.00000005	0	0.00000001	2.27813E-15	0.00000024	0.00227718	0

*f*_20_(*x*)	Best	-3.32199431	-3.32199517	-3.32199432	-3.32199517	-3.32199514	-3.16816134	-3.32199517
	Mean	-3.23885032	-3.32199517	-3.27443705	-3.31604271	-3.24969715	-3.02251139	-3.32199517
	Worst	-3.08390118	-3.32199517	-3.20310148	-3.20310205	-3.19844899	-2.62970467	-3.32199517
	Std	0.07507732	0	0.06512014	2.6583E-02	0.06602515	0.14476696	0

**Table 6:** The comparisons of t-test for *f*_1_ ~ *f*_20_.

Algorithm	*f*_1_(*x*)			*f*_2_(*x*)			*f*_3_(*x*)			*f*_4_(*x*)			*f*_5_(*x*)			*f*_6_(*x*)			*f*_7_(*x*)			*f*_8_(*x*)			*f*_9_(*x*)			*f*_10_(*x*)			
	H	P	Sig.	H	P	Sig.	H	P	Sig.	H	P	Sig.	H	P	Sig.	H	P	Sig.	H	P	Sig.	H	P	Sig.	H	P	Sig.	H	P	Sig.	
MSCA /PSO	0	1.000	+	0	1.000	+	0	1.000	+	0	1.000	+	0	0.999	+	0	1.000	+	0	1.000	+	0	1.000	+	0	1.000	+	0	1.000	+
MSCA /DE	0	1.000	+	0	1.000	+	0	1.000	+	0	1.000	+	0	1.000	+	NaN	NaN	≈	0	1.000	+	0	0.979	+	NaN	NaN	≈	0	1.000	+
MSCA /BA	0	1.000	+	0	0.980	+	0	1.000	+	0	1.000	+	0	0.999	+	0	1.000	+	0	1.000	+	0	1.000	+	0	1.000	+	0	1.000	+
MSCA /TLBO	NaN	NaN	≈	NaN	NaN	≈	0	1.000	+	NaN	NaN	≈	0	1.000	+	0	1.000	+	0	1.000	+	0	1.000	+	0	1.000	+	0	1.000	+
MSCA /GWO	NaN	NaN	≈	0	1.000	+	0	0.979	+	0	0.996	+	0	1.000	+	0	0.999	+	0	0.999	+	0	1.000	+	NaN	NaN	≈	0	1.000	+
MSCA /SCA	0	0.840	+	0	0.861	+	0	0.970	+	0	0.838	+	0	1.000	+	0	1.000	+	0	0.237	+	0	1.000	+	NaN	NaN	≈	0	0.935	+

Algorithm	*f*_11_(*x*)			*f*_12_(*x*)			*f*_13_(*x*)			*f*_14_(*x*)			*f*_15_(*x*)			*f*_16_(*x*)			*f*_17_(*x*)			*f*_18_(*x*)			*f*_19_(*x*)			*f*_20_(*x*)			number of winners
	H	P	Sig.	H	P	Sig.	H	P	Sig.	H		Sig.	H	P	Sig.	H	P	Sig.	H	P	Sig.	H	P	Sig.	H	P	Sig.	H	P	Sig.	

MSCA /PSO	0	0.999	+	0	1.000	+	0	1.000	+	0	20	+	0	0.976	+	0	0.999	+	0	0.999	+	0	0.999	+	0	0.999	+	0	1.000	+	20
MSCA /DE	NaN	NaN	≈	0	0.314	+	NaN	NaN	≈	NaN	13	≈	0	1.000	+	NaN	NaN	≈	0	0.500	+	1	2e-4	-	0	0.162	+	0	0.500	+	13
MSCA /BA	0	1.000	+	0	0.996	+	0	1.000	+	0	19	+	0	1.000	+	NaN	NaN	≈	0	0.500	+	1	0.001	-	0	1.000	+	0	0.999	+	19
MSCA /TLBO	NaN	NaN	≈	0	0.999	+	0	0.979	+	NaN	12	≈	NaN	NaN	≈	NaN	NaN	≈	0	0.500	+	1	2e-4	-	0	0.500	+	0	0.500	+	12
MSCA /GWO	NaN	NaN	≈	0	1.000	+	0	1.000	+	NaN	15	≈	0	0.979	+	NaN	NaN	≈	0	0.979	+	0	0.933	+	0	0.987	+	0	1.000	+	15
MSCA /SCA	NaN	NaN	≈	0	1.000	+	0	1.000	+	NaN	17	≈	0	0.942	+	0	1.000	+	0	0.999	+	0	0.997	+	0	1.000	+	0	1.000	+	17

**Table 7:** Test statistical results with different strategies.

Algorithm		*f*_1_(*x*)	*f*_3_(*x*)	*f*_5_(*x*)	*f*_7_(*x*)	*f*_9_(*x*)
SCA	Best	2.41227653E-99	4.35895925E-52	26.23629998	2.03984969E-06	0
	Mean	7.79692173E-86	4.66854445E-46	27.08121170	1.75661500E-05	0
	Worst	1.54558620E-84	4.16089002E-45	28.65013850	5.33965433E-05	0
	Std	3.45449900E-85	1.08000440E-45	0.68246666	1.42228474E-05	0

C-SCA	Best	3.10050150E-187	6.28523597E-119	26.40050047	2.76012374E-06	0
	Mean	3.07519823E-173	6.04561631E-105	27.03944045	2.34677075E-05	0
	Worst	3.73083365E-172	1.20899644E-103	28.04482481	1.15810162E-04	0
	Std	0	2.70338330E-104	0.55108210	2.52889873E-05	0

N-SCA	Best	1.32804165E-98	8.06533668E-57	25.94560968	8.07111724E-07	0
	Mean	1.14472110E-87	2.02781396E-44	26.90664795	1.80576054E-05	0
	Worst	2.23304334E-86	2.97707622E-43	28.65835507	8.44166161E-05	0
	Std	4.98734410E-87	6.76072976E-44	0.68607944	1.91972975E-05	0

G-SCA	Best	1.53038991E-247	6.21923122E-69	25.07435867	9.33982351E-11	0
	Mean	1.38742519E-228	1.57837645E-56	25.43773646	2.82512818E-10	0
	Worst	2.75931538E-227	2.15594380E-55	25.96615750	4.971578236E-9	0
	Std	0	5.19044381E-56	0.22877237	1.12630342E-10	0

MSCA	Best	0	0	7.76116677E-12	5.95321712E-07	0
	Mean	0	0	5.82095129E-06	2.06970501E-05	0
	Worst	0	0	2.47408894E-05	5.50713991E-05	0
	Std	0	0	6.12299693E-11	1.31769905E-05	0

Algorithm		*f*_11_(*x*)	*f*_13_(*x*)	*f*_15_(*x*)	*f*_17_(*x*)	*f*_19_(*x*)

SCA	Best	0	1.82820415	0.00031054	0.39788838	-3.86226093
	Mean	0	2.03922706	0.00041204	0.39799018	-3.85557758
	Worst	0	2.22177498	0.00126659	0.39837908	-3.85462395
	Std	0	0.10672949	0.00029067	0.00013970	0.00227718

C-SCA	Best	0	1.84451065	3.09612692E-04	0.39789121	-3.86272470
	Mean	0	2.05252816	3.62441048E-04	0.39795214	-3.85598742
	Worst	0	2.27441394	1.23237237E-03	0.39808342	-3.85454062
	Std	0	0.09517819	2.04871436E-04	0.00006120	0.00279692

N-SCA	Best	0	1.71770711	0.00030866	0.39789254	-3.86269936
	Mean	0	1.99159860	0.00037283	0.39799489	-3.85705467
	Worst	0	2.18349306	0.00124886	0.39841067	-3.85470429
	Std	0	0.11810434	0.00020886	0.00015085	0.00349793

G-SCA	Best	0	0.00384498	0.00030749	0.39788736	-3.86278215
	Mean	0	0.01677159	0.00046474	0.39788736	-3.86278199
	Worst	0	0.07222708	0.00122317	0.39788736	-3.86278146
	Std	0	0.01375995	0.00030237	0	0.00000021

MSCA	Best	0	1.349783804E-32	0.00030748598	0.397887358	-3.86278215
	Mean	0	1.349783804E-32	0.00030748598	0.397887358	-3.86278215
	Worst	0	1.349783804E-32	0.00030748598	0.397887358	-3.86278215
	Std	0	2.808011502E-48	0	0	0

**Table 8:** Test statistical results of Wilcoxon rank sum test.

Function	C-SCA /SCA		N-SCA /SCA		G-SCA /SCA	
	P value	Sig.	P value	Sig.	P value	Sig.
*f*_1_(*x*)	6.7956e-08	**+**	0.0909	≈	6.7956e-08	**+**
*f*_3_(*x*)	5.8923e-08	**+**	0.6359	≈	6.7956e-08	**+**
*f*_5_(*x*)	0.6554	≈	0.3369	≈	6.7956e-08	**+**
*f*_7_(*x*)	0.4094	≈	0.7557	≈	6.7956e-08	**+**
*f*_9_(*x*)	NaN	≈	NaN	≈	NaN	≈
*f*_11_(*x*)	NaN	≈	NaN	≈	NaN	≈
*f*_13_(*x*)	2.6898e-06	+	0.2184	≈	6.7956e-08	**+**
*f*_15_(*x*)	0.4570	≈	0.7972	≈	0.0123	**+**
*f*_17_(*x*)	0.6168	≈	0.9461	≈	8.0065e-09	**+**
*f*_19_(*x*)	0.3942	≈	0.7972	≈	5.3656e-08	**+**
number of winners (+/≈)		3/7	0/0		8/2	

**Table 9:** Statistical results for different values of *λ*.

Function	*λ* = 0.005 (mean)	*λ* = 0.01 ( mean)	*λ* = 0.02 ( mean)	*λ* = 0.03 ( mean)	*λ* = 0.05 ( mean)
*f*_2_(*x*)	1.46279E-57	6.15031E-58	2.04298E-57	5.87904E-57	**2.34908E-58(+)**
*f*_4_(*x*)	2.45329E-29	**3.29394E-30(+)**	1.00414E-29	7.65892E-30	2.45329E-29
*f*_6_(*x*)	3.68341	**3.63003(+)**	3.68868	3.72788	3.63373
*f*_8_(*x*)	-4363.10086	-4312.00222	**-4446.01569(+)**	-4410.44537	-4371.62707
*f*_10_(*x*)	0.41008	0.08565	**0.03147(+)**	0.37909	0.07129
*f*_12_(*x*)	**0.34002(+)**	0.35838	0.34005	0.34630	0.33576
*f*_14_(*x*)	0.99800	0.99800	0.99800	0.99800	0.99800
*f*_16_(*x*)	-1.03163	-1.03163	-1.03163	-1.03163	-1.03163
*f*_18_(*x*)	3.00000	3.00000	3.00000	3.00000	3.00000
*f*_20_(*x*)	-3.03411	**-3.07592(+)**	-3.05259	-3.04242	-3.03034
number of winners	1	3	2	0	1

**Table 10:** Statistical results for different values of *ε*.

Function	*ε* = 10 (mean)	*ε* = 30 ( mean)	*ε* = 60 ( mean)	*ε* = 90 ( mean)
*f*_2_(*x*)	2.1934E-150	**1.7432E-152(+)**	1.5276E-150	9.7415E-152
*f*_4_(*x*)	6.19395E-36	5.81864E-37	9.3441E-38	**2.0556E-38(+)**
*f*_6_(*x*)	0.00152	**0.00148(+) **	0.00151	0.00153
*f*_8_(*x*)	-7566.98206	**-7598.33875(+) **	-7561.27736	-7596.35448
*f*_10_(*x*)	**0.198701(+) **	0.398512	0.398299	0.398304
*f*_12_(*x*)	0.00026	**0.00024(+) **	0.00027	0.00025
*f*_14_(*x*)	0.99800	0.99800	0.99800	0.99800
*f*_16_(*x*)	-1.03163	-1.03163	-1.03163	-1.03163
*f*_18_(*x*)	3.00000	3.00000	3.00000	3.00000
*f*_20_(*x*)	-3.25517	**-3.23962(+) **	-3.23028	-3.23419
number of winners	1	5	0	1

## Data Availability

All data are included within the tables and figures of this article.
